# Fatigue Assessment by Blink Detected with Attachable Optical Sensors of Dye-Sensitized Photovoltaic Cells

**DOI:** 10.3390/mi9060310

**Published:** 2018-06-20

**Authors:** Ryogo Horiuchi, Tomohito Ogasawara, Norihisa Miki

**Affiliations:** Department of Mechanical Engineering, Keio University, 3-14-1 Hiyoshi, Kohoku-ku, Yokohama, Kanagawa 223-8522, Japan; jjryogo747@gmail.com (R.H.); tomo.0602.72003@gmail.com (T.O.)

**Keywords:** fatigue, dye-sensitized photovoltaic cells, wearable, blink, sensors, micro/nano technology, microfabrication

## Abstract

This paper demonstrates fatigue assessment based on eye blinks that are detected by dye-sensitized photovoltaic cells. In particular, the sensors were attached to the temple of eyeglasses and positioned at the lateral side of the eye. They are wearable, did not majorly disturb the user’s eyesight, and detected the position of the eyelid or the eye state. The optimal location of the sensor was experimentally investigated by evaluating the detection accuracy of blinks. We conducted fatigue assessment experiments using the developed wearable system, or smart glasses. Several parameters, including the frequency, duration, and velocity of eye blinks, were extracted as fatigue indices. Successful fatigue assessment by the proposed system will be of great benefit for maximizing performance and maintenance of physical/mental health.

## 1. Introduction

Fatigue assessment is crucial to secure safety and efficiency in operation. For such applications, the assessment system itself should provide the users’ minimum physical and mental stress; the whole system should be light enough to be wearable, and should not disturb the users’ activities and eyesight. Real-time process is also an important requirement, which encourages us to discover fatigue indices that can be easily measured as well as processed.

Heart rate variance (HRV) uses an R-R interval, or RRI, of an electrocardiogram (ECG). The RRI is deduced from the measured ECG and Fourier transformed to calculate the autonomic nerve index as the power ratio of the low-frequency (0.05–0.15 Hz) and high-frequency (0.15–0.40 Hz) bands. Heart rate variance increases with fatigue and decreases during recovery [1,2].

Electroencephalograms (EEGs), or brain waves, are reported to represent fatigue [3,4,5,6]. The relationship between EEG and mental fatigue has been explored by many reports, where they attempted to find effective indexes of fatigue from EEGs. Four-frequency bands were often used, including alpha (8–13 Hz), beta (17–34 Hz), theta (4–8 Hz), and delta (0.5–4 Hz). The peak frequency of the alpha power decreases with loaded mental work and increases with resting [3]. The ratio of the low alpha (8–10.5 Hz) to the high alpha (10.5–13 Hz) was experimentally verified to better represent the mental work load [4]. The other bands, beta, theta, and a combination of all the bands, were correlated to fatigue [5,6,7,8,9,10].

**Figure 1:**
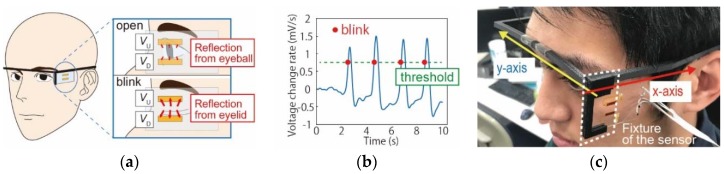
Smart glass system to detect blinks. (**a**) Schematic view of the sensor cells. Two dye-sensitized photovoltaic cells were patterned on a glass substrate as the optical sensors, which detect the reflection light from the eye. The reflection light from the eyelid when the subject blinks is larger than that from the eyeball when he/she opens the eye. (**b**) Detection of the blink. When the derivative of the average output of the two sensor cells exceeds the threshold, we consider the subject to be blinking. (**c**) Position of the system. The positions in the *x*- and *y*-axes can be varied by the location to set the fixture onto the temple and by the holder design, respectively.

Information acquired from eyes, including movement of the eye and eye blinks, provides the state of the subjects [11,12,13]. In order to obtain the information with minimal physical and mental stress, we used a wearable, see-through eyeglass-type eye-tracking system [14,15]. The system had an array of transparent optical sensors, which were dye-sensitized photovoltaic devices [16,17,18]. While dye-sensitized photovoltaic devices have been studied as next generation solar cells, we utilized them as optical sensors. In this context, the sensor property we were most interested in was the detection accuracy of eye movement and eye blinks. The properties of the dye-sensitized photovoltaic devices, such as photo conversion efficiency, wavelength dependence, etc., are not discussed. The sensor system was wearable and light, and did not have cameras pointing at the user, and therefore, provided little physical and mental stress. In our prior work, the indices related to eye blinks were found to reflect the fatigue of the subjects [15]. In this work, in order to further reduce stress to the users, we designed and fabricated a system to detect eye blinks, which can be attached to the temple of the eyeglasses and positioned at the lateral side of the face, as shown in Figure 1. It does not hinder the eyesight of the users, and is not affected by the movement of the eye. It can be attached to various types of eyeglasses and eyeglass-type devices. We firstly deduced the optimal position of the blink detection system experimentally and then attempted to correlate the users’ fatigue with the eye blinks that were detected by the system.

## 2. Materials and Methods

### 2.1. Blink Detection System

The blink detection system is composed of two dye-sensitized photoelectric cells, as shown in Figure 1. The cell is patterned on a glass substrate 2 mm in width and 8 mm in length. The detailed fabrication processes were described in prior work [17] and in Figure S1 in the Supplemental Material. Indium-Tin~Oxide (ITO) thin film on the glass substrate was fine-patterned to form an electrical circuit. A titanium dioxide nanoparticles film was patterned to form the cathode, on which ruthenium dyes adsorb. Fabrication processes of the cells were completed with encapsulation of the electrolyte between the cathode and the anode on the glass substrates. The fabricated cell was attached to the temple of the eyeglasses. The sensors detect the reflection light from the eyelid and the eyeball. The reflection light from the eyeball is weaker than that from the eyelid. Thus, the sensors can detect the eye-state including blinks.

In the blink detection, the derivative of the average of the output voltages of the two cells (*V_U_* and *V_D_*) is used, as shown in Figure 1a. Blink is detected when the derivative with respect to time, or voltage change rate, is greater than the threshold. In our prior work, we investigated the successful detection of the eye blinks with respect to the threshold. Based on the experimental results, we set the threshold to be 70% of the maximum voltage change rate [14,15], as illustrated in Figure 1b. The optimal position of the cells to have the most reliable detection was experimentally deduced. The position of the system in *x*- and *y*-axes were varied, with the position of the fixture at the temple and the fixture design, as shown in Figure 1c. Four subjects (21~24 years old, 3 males and 1 female) were requested to wear the smart glass system and blink every 2 s. The number of blinks detected by the system was compared to that obtained by an external camera. The average of the voltage change rate at the blinks was defined as the detection sensitivity (mV/s) and used to find the optimal position of the system. Each subject conducted the experiments four times.

### 2.2. Fatigue Assessment Experiments

The experiments described in this work were approved by the research ethics committee of the Faculty of Science and Technology, Keio University. Sixteen subjects (aged 21~24, 13 males and 3 females) participated in the experiments.

The subjects were directed to perform the Uchida–Kraepelin (U–K) psychodiagnostic test, which was originally developed by Uchida [19]. The U–K test is based on a series of simple addition tasks and measures a subject’s ability to perform tasks quickly and accurately. It is also used as a simple but efficient mental stressor.

We attempted to assess the fatigue by three methods: subjective fatigue symptoms or “jikaku-sho shirabe” in Japanese, HRV, and the blinks. Subjective fatigue symptoms were deduced by a questionnaire for 25 symptoms [20,21,22]. The subjects were requested to score 1–5 for each symptom, where 1 is totally disagree and 5 is totally agree. This protocol was proposed by the Industrial Fatigue Research Committee of Japanese Occupational Health in 2002. The symptoms are categorized into five factors, which are (i) drowsiness, (ii) instability, (iii) uneasiness, (iv) local pain or dullness, and (v) eyestrain. These factors were correlated with the number of U–K tests.

Heart rate variability was measured with flat electrodes from the arm and leg of the subject and then acquired by a polygraph. The signals were processed by Labchart (ADInstruments, Nagoya, Japan), where power supply noise at 50 Hz was cut and signals from 0.5 to 10 Hz were passed. In the power spectrum analysis of HRV, the ratio of the low-frequency power to the high-frequency power was deduced to objectively and quantitatively assess the fatigue induced by the U–K tests.

Blinks by the subjects were measured using the smart glass system. We investigated (i) the number of blinks, (ii) the number of blink bursts, (iii) the blink burst rate, (iv) the blink duration, and (v) blink velocity as the candidates for the fatigue indices. The blink bursts were series of blinks between 0.5~2.0 s. The blink duration represents the period while the eyes are closed. The blink velocity is the velocity of the eyelids. All the candidates can be deduced from the output signals of the smart glass system.

In analyses of subjective fatigue symptoms, HRV, and the blinks, the obtained data were normalized as follows.

(1) $$\;z_{i}=\frac{x_{i}-\tilde{x}}{\sigma^{2}}$$

where $x_{i}$ is the acquired datum, $\tilde{x}$ is the average, and $\sigma^{2}$ is the variance. The data group has an average of 0 and a standard deviation of 1.

**Figure 2:**
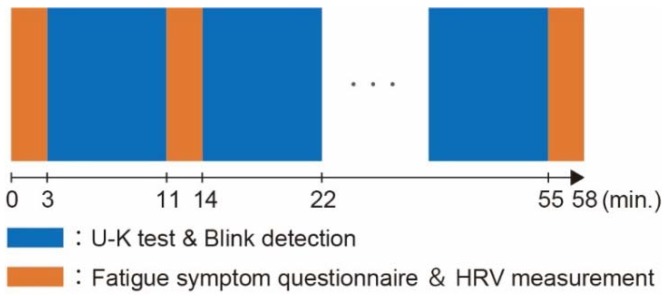
Protocol of the fatigue assessment experiments.

The protocol of the fatigue assessment experiments is illustrated in Figure 2. The fatigue symptom questionnaire and HRV measurement were conducted for 3 min, which was followed by U–K tests and blink detection for 8 min. This set was iterated five times, and the series of experiments ended with another fatigue symptom questionnaire and HRV measurement.

The obtained data are not likely to be normally distributed. We conducted Steel–Dwass multiple comparison tests to investigate the indices with respect to the number of U–K tests or fatigue.

## 3. Results

### 3.1. Optimal Position of the Blink Detection System

**Figure 3:**
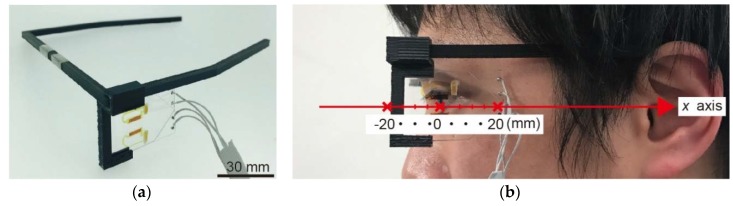
(**a**) Photo of the fabricated smart glass system. (**b**) Photo of the experiments highlighting the position of the system at the *x*-axis.

Figure 3a shows the photo of the fabricated smart glass system. Two dye-sensitized photovoltaic cells are formed on a glass substrate as the optical sensors, which is attached to the temple of the eyeglasses, which is suitable for wearable applications. The position of the glass substrate can be varied in *x*- and *y*-axes as shown in Figure 1c and Figure 3b. The position lateral to the pupil is set to be *x* = 0.

**Figure 4:**
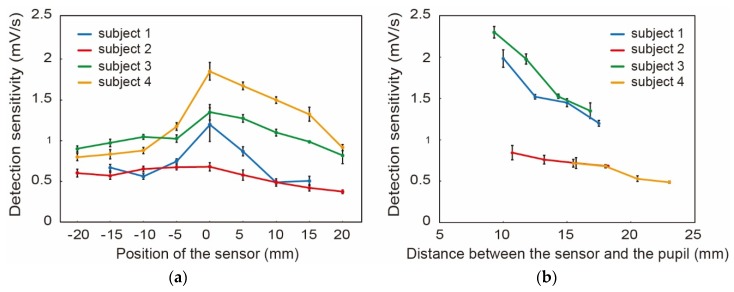
Detection sensitivities with respect to the sensor position at the (**a**) *x*-axis and (**b**) *y*-axis. Four subjects conducted experiments four times for each condition. The optimal position on the *x*-axis was found to be *x* = 0 (i.e., the lateral to the eye). On the *y*-axis, the sensitivity increased as the sensor was located closer to the eye. The error bars represent the standard deviation.

The detection sensitivity, which is the derivative of the voltage at the blinks, with respect to *x*-axis position and *y*-axis position is shown for four subjects in Figure 4a,b, respectively. At the *x*-axis, for all the subjects, the sensitivity showed its maximum at *x* = 0. This is reasonable since the motion of the eyelid is the largest at *x* = 0. We were concerned about the effect of eyelashes, however, it did not appear in the experiments. Differences among the subjects in absolute values of the sensitivity were observed, which were calibrated in the following blink detection experiments.

At the *y*-axis, the sensitivity increased as the sensor was closer to the eye. Since the sensor detects the scattered light from the eyelid, the sensor output becomes larger as the gap between the sensor and eyelid decreases. In the following experiments, we set the smart glass system such that the distance from the eye was as small as possible while the sensor did not touch the lateral side of the head.

**Figure 5:**
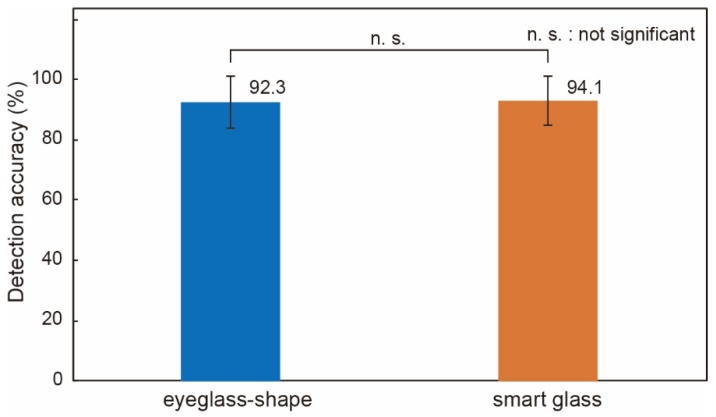
Blink detection accuracy. The results obtained by the smart glass system were compared to the eyeglass system in our prior work and showed no significant difference. Four subjects participated in the experiments for each device.

The blink detection accuracy was deduced by the ratio of the blinks detected by the smart glass system to those detected by an external video camera image. The experiments were conducted for four subjects, and the average and standard deviation is shown in Figure 5. It was found to be as high as 94% and showed no significant difference (*p*-value > 0.05) from our previous device [15].

### 3.2. Fatigue Assessment Experiments

#### 3.2.1. Subjective Fatigue Symptoms

**Figure 6:**
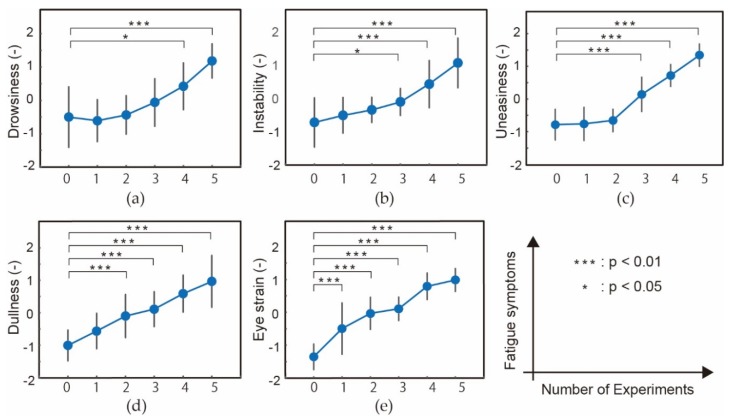
Normalized (**a**) drowsiness, (**b**) instability, (**c**) uneasiness, (**d**) local pain or dullness, and (**e**) eyestrain with respect to the number of U–K tests (*n* = 16). “*p*” represents the *p*-value in statistics hypothesis testing.

Figure 6 shows the normalized results of (a) drowsiness, (b) instability, (c) uneasiness, (d) local pain or dullness, and (e) eyestrain, with respect to the number of U–K tests. For all the symptoms, significant differences between before (experiment number 0) and after all the U–K tests (number 5) were obtained. The figures also show significant differences in the results at each experiment from before the U–K tests, if any. The results indicate that the applied U–K tests induced fatigue to the subjects, which increased with the number of tests.

Of the five symptoms, eye strain appears to correlate with the number of experiments most. Eye strain increases with the number of tests from the first to the final test. Drowsiness, instability, and uneasiness increased from the third test. Since fatigue is the summary of these symptoms, we cannot say that fatigue increases with the number of tests almost linearly, as shown in the eye strain, or fatigue does not appear in the first two tests and starts increasing from the third test. However, at least we can conclude that fatigue increased as the U–K tests were iterated, and this trend was in common among all 16 subjects with relatively small deviation. It is reasonable to presume fatigue accumulates from the beginning of the tests, although it may be little. If we would like to detect such small amounts of fatigue, eye strain is the most suitable symptom to investigate, which can be represented by the blinks.

#### 3.2.2. HRV

**Figure 7:**
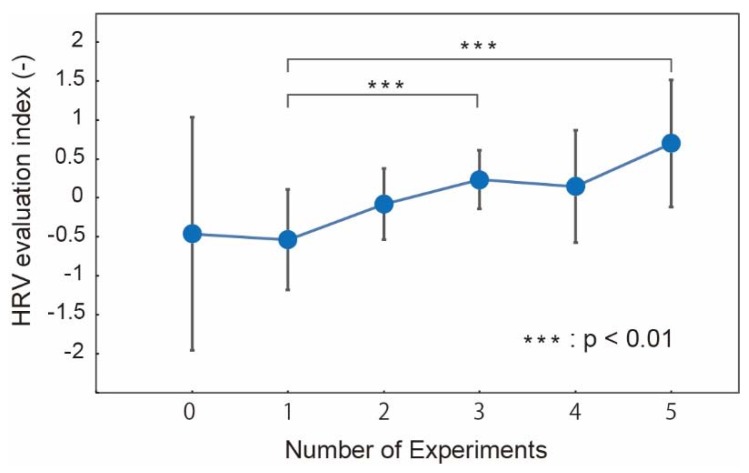
Normalized heart rate variance (HRV) with respect to the number of U–K tests (*n* = 16). “*p*” represents the *p*-value in statistics hypothesis testing.

Figure 7 shows the normalized HRV with respect to the number of U–K tests. Large variations among the subjects in HRV before the U–K tests was found. As an overall trend, HRV increased with the number of tests. Significant differences were found between after the first test and the final test. However, the trend appears to be less definitive than the subjective evaluation, as shown in Figure 6. In our other work [5], HRV did not match well with the subjective evaluation, either. Heart rate variance may provide qualitative information on fatigue, but it is not suitable to quantitatively assess fatigue.

#### 3.2.3. Blinks

**Figure 8:**
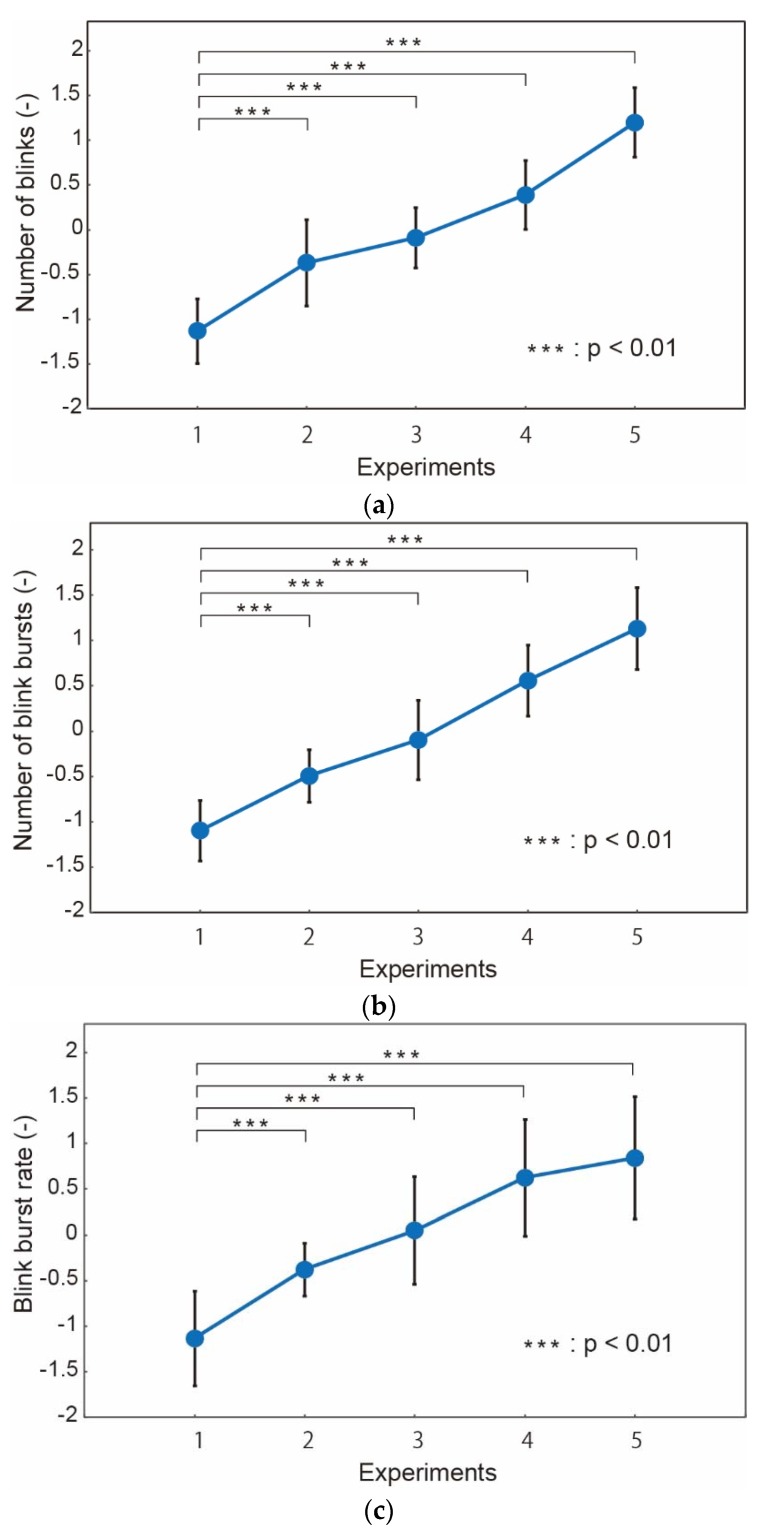

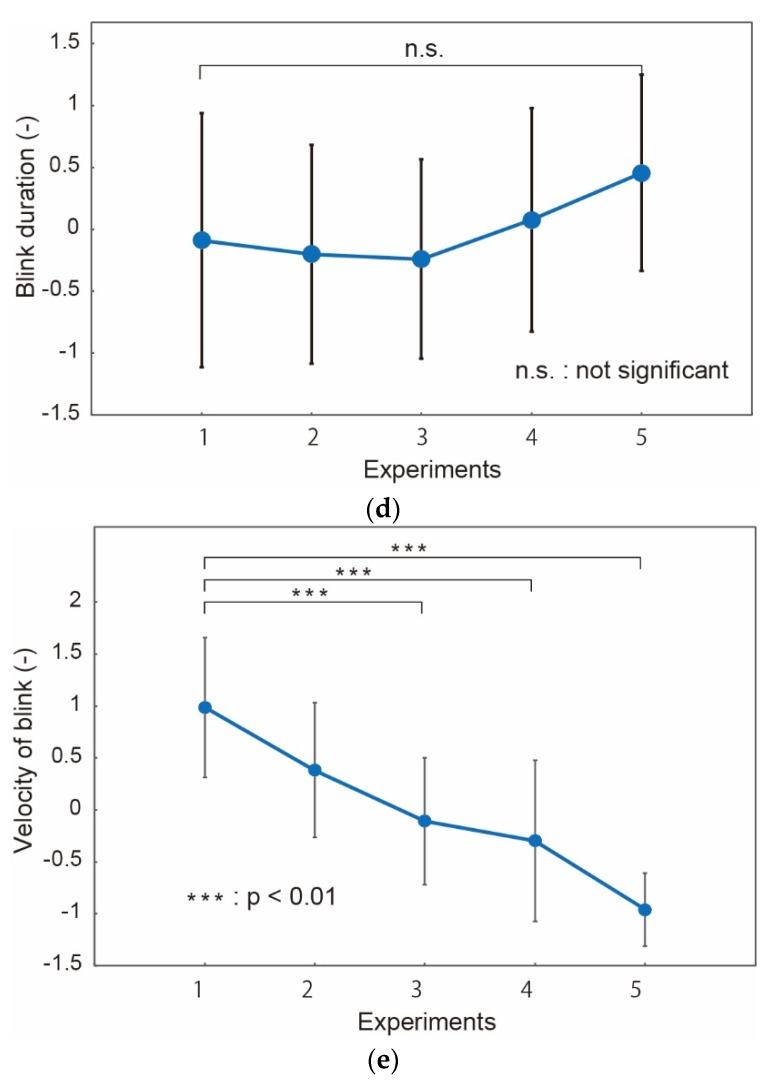
Obtained (**a**) the number of blinks, (**b**) the number of blink bursts, (**c**) the blink burst rate, (**d**) the blink duration, and (**e**) blink velocity with respect to the number of U–K tests. “*p*” represents the *p*-value in statistics hypothesis testing.

We investigated (i) the number of blinks, (ii) the number of blink bursts, (iii) the blink burst rate, (iv) the blink duration, and (v) blink velocity as candidates for the fatigue indices. The results are shown in Figure 8.

The number of blinks increased monotonously with the number of tests, as shown in Figure 8a. Significant difference (*p* < 0.01) was found even between the 1st and 2nd tests. The trend is similar with the eye strain (Figure 6e). Given this agreement and rather easy measurement, the number of blinks is a strong candidate for the fatigue index.

As shown in Figure 8b,c, blink bursts showed good correlation with the number of blinks. A significant difference was found from the 2nd tests, which represents the high sensitivity with fatigue similarly with the number of blinks.

**Figure 9:**
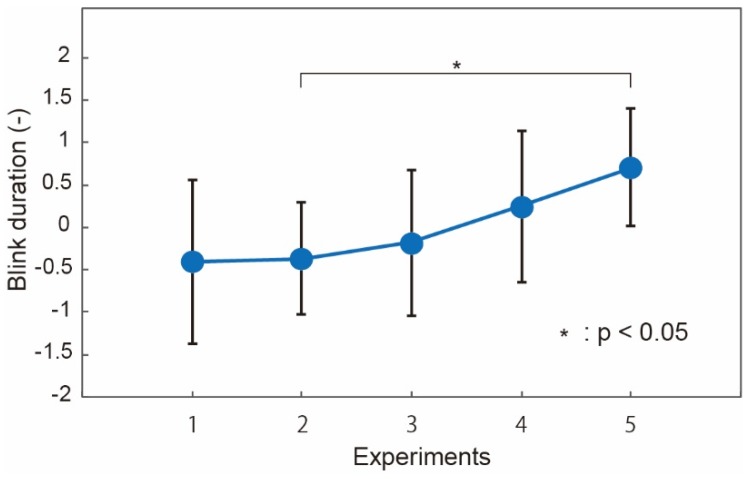
Blink duration for the subjects who did not have a low level of awakeness. “*p*” represents the *p*-value in statistics hypothesis testing.

Blink duration, on the contrary, did not show good correlation with the number of the U–K tests or fatigue, as shown in Figure 8d. The duration was reported as a fatigue index in prior work [23]. It was also reported that the duration did not extend when the level of awakeness was too low. We considered that some subjects might have a low level of awakeness during the tests. The level of awakeness can be evaluated by the activity of the parasympathica divisionis, which is reflected in the high frequency component of the heart rate. We investigated the blink duration of the subjects whose high frequency component of the heart rate did not increase. The results are shown in Figure 9. The blink duration increased as a trend. However, we consider that the blink duration cannot be a good fatigue index because of the low correlation with the fatigue and limitation of the subjects.

Figure 8e shows the normalized blink velocity with respect to the number of tests. The velocity decreased with the tests. A significant difference was found between the 1st and 3rd test. However, the variation among the subjects was found to be larger and the correlation with the number of tests was smaller than the number of blinks and blink bursts.

## 4. Discussion

The number of blinks was found to be the most suitable index to assess fatigue. However, note that it is reasonable to presume the number of blinks will hit its maximum value while fatigue can keep increasing. In our experiments, the tests were conducted for approximately one hour. For this fatigue level, the number of blinks is a good index. In our future work, we will investigate the range of fatigue level, where the number of blinks maintains good correlation with fatigue.

**Figure 10:**
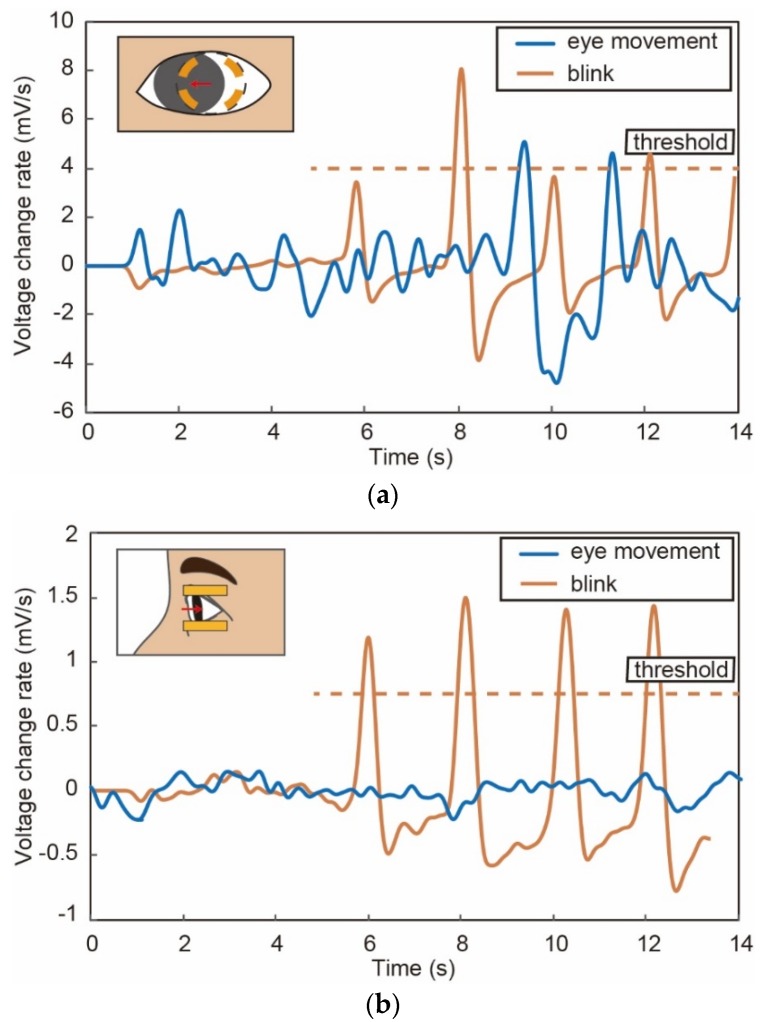
The sensor outputs when the subject moves the eye and blinks while moving the eye in case of (**a**) eyeglasses type device and (**b**) smart glass device.

Table 1 summarizes the results obtained in our prior work with an eyeglass-type system [15] and this work with the smart glass. The indices obtained by the smart glass system were found to be better than those obtained by the eyeglass system. As we showed in Figure 5, the blink detection accuracy was found to be comparable. However, during the U–K tests, the subjects move their eyes, which affects the detection accuracy more dominantly for the eyeglass type than the smart glasses. Figure 10 shows the sensor output data when the subject moves his/her eye with an angle of 10° and blinks with the eye movement. Obviously, detection of blinks with the eyeglasses-type device is disturbed by the eye movement. We conclude that the smart glass system proposed herein is more suitable to assess fatigue than our previous device. 

## 5. Conclusions

Fatigue assessment using the proposed sensor system was successfully demonstrated. The number of blinks and blink bursts showed good correlation with the number of U–K tests, which also showed good agreement with the fatigue symptoms that were deduced from subjective questionnaires. The measurement of the number of blinks can be achieved via simple processes, which can contribute to real-time fatigue assessment. Heart rate variability, or HRV, showed less correlation with the tests. It was experimentally found that the sensor system attached to the temple of the eyeglasses, or smart glass system, was more suitable than the eyeglasses-type system due to the little disturbance from eye movement. Fatigue assessment by the proposed smart glass system is of great benefit for maximizing performance and maintenance of physical/mental health.

## Figures and Tables

**Table 1:** Summary of the candidates for fatigue index.

Blink Detection Sensor	Number of Blinks	Number of Blink Bursts	Blink Bursts Rate	Blink Duration	Velocity of Blinks
Eyeglasses Type [15]	†	†	†	n.s.	--
Smart Glass Type	****	****	****	†	****

Notes: ****: *p* < 0.001; †: 0.05 < *p* < 0.1; n.s.: not significant.

## Supplementary Materials

The following are available online at http://www.mdpi.com/2072-666X/9/6/310/s1.
