# Supplementary material for: Fatigue Assessment by Blink Detected with Attachable Optical Sensors of Dye-Sensitized Photovoltaic Cells
Source: Micromachines (Basel). 2018 Jun 20;9(6):310. doi: 10.3390/mi9060310 (PMC6187843; doi:10.3390/mi9060310)
Supplement: Supplementary file 1 [file micromachines-09-00310-s001.pdf]

Supplementary Materials: Fatigue Assessment by Blink Detected with Attachable Optical Sensors of Dye-Sensitized Photovoltaic Cells

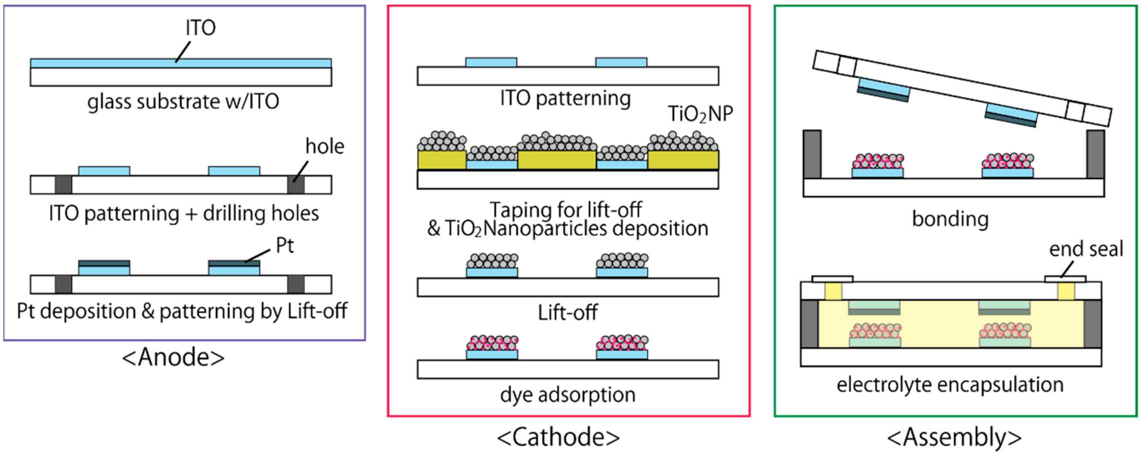

Figure S1. Fabrication process of the microfabricated dye-sensitized photovoltaic cells, which was used as the transparent optical sensors to detect eye blinks.
